# Quasi-Stationarity of EEG for Intraoperative Monitoring during Spinal Surgeries

**DOI:** 10.1155/2014/468269

**Published:** 2014-02-17

**Authors:** Krishnatej Vedala, S. M. Amin Motahari, Mohammed Goryawala, Mercedes Cabrerizo, Ilker Yaylali, Malek Adjouadi

**Affiliations:** ^1^Center for Advanced Technology and Education, FIU College of Engineering and Computing, Room Number EC2672, 10555 West Flagler Street, Miami, FL 33174, USA; ^2^Department of Clinical Neurophysiology, Oregon Health and Science University, Portland, OR 97239-3098, USA

## Abstract

We present a study and application of quasi-stationarity of electroencephalogram for intraoperative neurophysiological monitoring (IONM) and an application of Chebyshev time windowing for preconditioning SSEP trials to retain the morphological characteristics of somatosensory evoked potentials (SSEP). This preconditioning was followed by the application of a principal component analysis (PCA)-based algorithm utilizing quasi-stationarity of EEG on 12 preconditioned trials. This method is shown empirically to be more clinically viable than present day approaches. In all twelve cases, the algorithm takes 4 sec to extract an SSEP signal, as compared to conventional methods, which take several minutes. The monitoring process using the algorithm was successful and proved conclusive under the clinical constraints throughout the different surgical procedures with an accuracy of 91.5%. Higher accuracy and faster execution time, observed in the present study, in determining the SSEP signals provide a much improved and effective neurophysiological monitoring process.

## 1. Introduction

Sensory evoked potentials (SEP) are the signals that are observed in the brain due to an applied stimulus. When the stimulus is applied to the somatic sensory neuron, it is termed as somatosensory evoked potential (SSEP) [1, 2]. The value of these SSEP is important during neurosurgeries wherein the SSEP travels from the sensory neuron through the spinal cord to the brain. The SSEP is recorded and noted prior to the surgical procedure and are expected to remain consistent throughout the procedure [3]. Hence, a discrepancy in the SSEP is a cause of alarm for the concerned neurosurgeons informing a possible dysfunction along the nervous pathway. This fact is utilized for the early detection of potential changes leading to spinal cord dysfunction. The surgeons can then adopt the necessary protocols to prevent potential neurophysiological defects. Coupled with the fact that this is a noninvasive technique, the SSEP monitoring has become an inherent part of intraoperative neurophysiological monitoring (IONM). The SSEP recordings from cortical electrodes are buried deep within the ongoing EEG signals and surrounding equipment noise. The fact that SSEP remains consistent was used to implement a signal averaging method to extract the SSEP [4, 5]. This method, however, has the major disadvantage of requiring a large number of trials to be averaged, typically of the order of 200–5000, which need few minutes to process.

The primary objective of the present study was in providing the ability to extract the SSEP signal using a minimal number of trials. Earlier workers have proposed methods like parametric modeling [6–8], adaptive filtering [9–11], and time frequency analysis to obtain SSEP with minimum trials [6, 10, 12, 13]. Nonetheless, these methods still pose different constraints and contend with different limitations in extracting the SSEP accurately, consistently, and with the appropriate morphology. For example, in an earlier study [14], we proposed the use of the AMUSE algorithm followed by an infinite impulse response (IIR) filter and a unique application of the Walsh transform to automate the SSEP detection. Although the accuracy and time consistency were achieved with a high degree, the method did not reveal the true nature of the morphological characteristics of the SSEP. This limitation is due to the fact that with a minimal number of unfiltered trials that were fed to the algorithm, it was very difficult to recover the true morphology of the SSEP signal, although accurately detected by the Walsh transform.

For these reasons, the proposed study presents a comprehensive and fast approach to overcome such limitations, with an initial first step of preprocessing the raw signals using a Chebyshev time window. This method, its validation, implementation results, and the improvement achieved therein are reported in the present communication.

## 2. Methods

### 2.1. Ethics Statement

The experimental work of this study was approved by the Office of Research Integrity, Florida International University, Miami; the approval numbers are 052708-03 and 100410-00. The data was collected as a routine part of a spine surgery and deidentified at the source, thus protecting the human subjects. A written consent was not obtained from the patients and waived by IRB number 104100-00 since no information that identifies the individuals was stored.

### 2.2. Data Acquisition

The programming applications Matlab (The Mathworks Inc., Natick, Massachusetts, USA) and GNU Octave (http://www.octave.org/) were used to implement the algorithm explained below.

**Figure 1:**
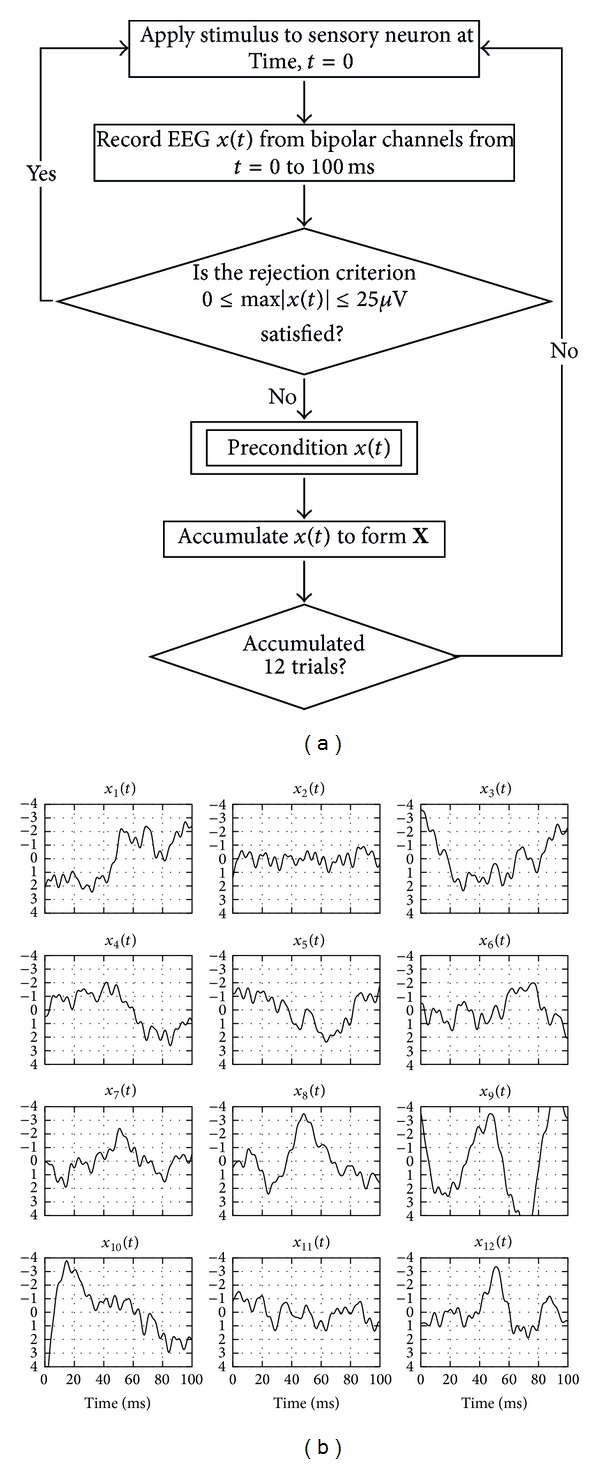
Diagram for “data acquisition” inclusive of the rejection criterion: (a) algorithm flowchart for data acquisition; (b) one such resulting matrix consisting of 12 satisfactory trials for patient 8 from Table 1.

- Stimulus of intensity 45 mV was applied to the posterior tibial nerve at a rate of 3.1 Hz.
- The corresponding cortical response was recorded simultaneously via *C*_3_-*C*_4_ and *C*_*Z*_-*F*_*Z*_ bipolar channels for a duration of 100 ms. The sampling rate of the recordings was 6400 Hz and thus, the recordings had 640 time samples.
- In the clinical settings, it is common for the electrodes to get disturbed and the recordings become fraught with noise levels that are nonsensical or seem at time as if they are disconnected. Such trials can be identified and eliminated if (i) the amplitude is zero or (ii) the amplitude is higher than 25 V. This restriction criterion is in accordance with the established SSEP clinical guidelines and is routinely adopted [2]. Hence, in this study, the same criterion was adopted for bad trial rejection as illustrated in Figure 1.

For analysis, data was collected from 12 surgical procedures and analysed remotely as presented in Table 1. The surgeries were performed by trained neurosurgeons at Oregon Health and Science University, Portland, OR, USA. The IONM recording equipment used was Cascade Intraoperative Monitoring System (Cadwell, Kennetwick, MA). The data was collected as a routine part of a spine surgery and were de-identified at the source, thus protecting the human subjects. A written consent was not obtained from the patients and waived by IRB number 104100-00 since no information that identifies the individuals was stored.

### 2.3. Window-Based Signal Preconditioning

Each of the signals, as they are acquired, was first zero padded to move the SSEP to the middle of the window function where the tapering effect of filtering in frequency domain was minimal. We chose Chebyshev window as an improvement to our previously presented Gaussian template [15]. A Chebyshev window of the same length was applied to this signal. This has the effect of minimizing the Chebyshev norm of the side lobes while increasing the main lobe width on every FFT component that is to be calculated. This implies that the SSEP signal is concentrated in the main lobe ensuring the SSEP morphology is maintained in further processing. The signal is converted to frequency domain by FFT. In this domain, we applied rectangular passband window from 50 Hz to 120 Hz. The 60 Hz component was completely removed.

The inverse FFT of this signal is what is termed as the preconditioned signal when the zero padding is removed. Given the 640 time samples, the *n*th preconditioned signal can then be represented as follows:

(1) $$\begin{array}{c} x_{n}=x_{n}\left(t\right)=\left[\begin{array}{cccc} x_{n}\left(t_{0}\right) & x_{n}\left(t_{1}\right) & \cdots & x_{n}\left(t_{639}\right) \end{array}\right], \end{array}$$

where the time samples are uniformly distributed between 0 ms and 100 ms.

Once 12 such uncorrupted and preconditioned trials were obtained, they were ordered to form a 12 × 640 matrix **X**:

(2) $$\begin{array}{c} X=\left[\begin{array}{c} x_{1}\\ x_{2}\\ \vdots \\ x_{12} \end{array}\right]=\left[\begin{array}{cccc} x_{1}\left(t_{0}\right) & x_{1}\left(t_{1}\right) & \cdots & x_{1}\left(t_{639}\right)\\ x_{2}\left(t_{0}\right) & x_{2}\left(t_{1}\right) & \cdots & x_{2}\left(t_{639}\right)\\ \vdots & \vdots & \ddots & \vdots \\ x_{12}\left(t_{0}\right) & x_{12}\left(t_{1}\right) & \cdots & x_{12}\left(t_{639}\right) \end{array}\right]. \end{array}$$

**Figure 2:**
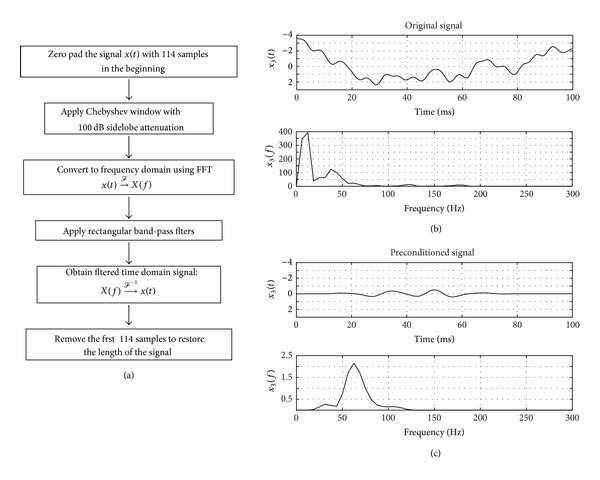
Diagram for “window based preconditioning.” (a) Algorithm flowchart for signal preconditioning. (b) The signal *x*_3_(*t*) before pre-conditioning from Figure 1(b) along with its power spectrum. (c) The result of preconditioning the signal in (b) and its corresponding power spectrum.

The window-based preconditioning, along with its specific steps, is illustrated in Figure 2.

### 2.4. Eigenspace Filtering

Algorithm for multiple signal extraction (AMUSE) is a principal component analysis (PCA) based algorithm intended for blind source separation [16]. It is equivalent to cascading two PCA systems with the following assumptions:

- signals in the data set are zero-mean wide sense ergodic processes, the components of which are mutually independent;
- noise in the data is zero-mean white Gaussian noise.

**Figure 3:**
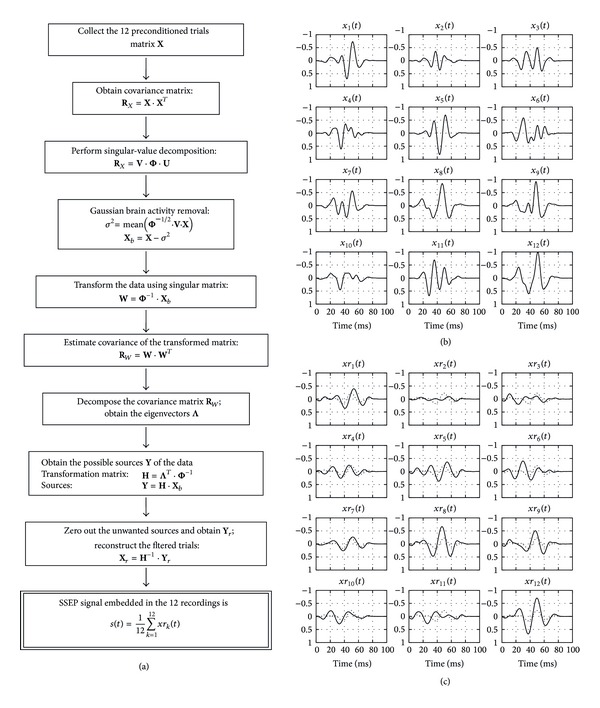
Diagram for “eigenspace filtering”. (a) Algorithm flowchart for eigenspace filtering. (b) Set of *x*_*k*_(*t*) signals from Figure 1(b) before eigen filtering. (c) Set of *xr*_*k*_(*t*) signals after filtering with the extracted SSEP signal, *s*(*t*), shown by dotted line superimposed on the signals for easy comparison.

The two assumptions were found to be valid for their application here because (i) the SSEP is a very small component of the recording and (ii) the major component is the background brain activity. This brain activity is what we need to eliminate in order to obtain the buried SSEP. The brain activity possesses the characteristic of of being zero-mean Gaussian process under anesthesia [17] and thus it satisfies the above condition (ii). For this, the assembly matrix **X** was used and implemented as follows (Figure 3).

#### 2.4.1. Covariance Matrix

The covariance matrix of **X** was obtained as

(3) $$\begin{array}{c} R_{X}=X\cdot X^{T}. \end{array}$$

Thus, the covariance matrix had the dimension 12 × 12.

#### 2.4.2. Singular Value Decomposition (SVD)

The covariance matrix **R**_**X**_ was then decomposed into singular values using the SVD algorithm [18]:

(4) $$\begin{array}{c} R_{X}=V\cdot \Phi \cdot U, \end{array}$$

where **V** is the matrix whose columns are left-singular vectors, Φ is the diagonal matrix whose elements are singular values or eigenvalues, and **U** is the matrix whose rows are right-singular vectors.

#### 2.4.3. Gaussian Brain Activity Removal

The variance of the Gaussian noise component, due to the brain activity surrounding the electrodes during the 12 recordings, was estimated from this decomposition:

(5) $$\begin{array}{c} \sigma^{2}=\text{mean}\;\left(\Phi^{-1/2}\cdot V\cdot X\right). \end{array}$$

Hence, elimination of this noise minimized the spatial source spread and ensured the components to be independent:

(6) $$\begin{array}{c} X_{b}=X-\sigma^{2}. \end{array}$$

#### 2.4.4. Intertrial Brain Activity Removal

The independent components of the remaining background brain activity were obtained by further decomposition following Sections 2.4.1 and 2.4.2 above.

(i) Transform the data using the singular matrix Φ:
  (7) $$\begin{array}{c} W=\Phi^{-1}\cdot X_{b}. \end{array}$$
(ii) Estimate the covariance matrix for **W**_*b*_:
  (8) $$\begin{array}{c} R_{W}=W\cdot W^{T}. \end{array}$$
(iii) Consider singular value decomposition of **R**_*W*_ and obtained Λ as its eigenvectors.
(iv) The possible sources (components) of the data were obtained by
  (9) $$\begin{array}{c} H=\Lambda^{T}\cdot \Phi^{-1},\;\;Y=H\cdot X_{b}. \end{array}$$
  In their structure, (i) the components were arranged in the descending order of their corresponding eigenvalues and (ii) the components that correspond to the eigenvalues, more than one standard deviation above the mean, are the components that contribute to the background brain activity.
(v) These components were identified and removed by zeroing out the components resulting in **Y**_*r*_.
(vi) The signals were reconstructed from **Y**_*r*_ with the noise contributing components removed:
  (10) $$\begin{array}{c} X_{r}=H^{-1}\cdot Y_{r}. \end{array}$$

#### 2.4.5. Reconstruction and SSEP

We claim that the **X**_*r*_ signals are the cleanest signals obtained from 12 trials because they are now devoid of

- undesired frequency components that are known a priori due to SSEP signal characteristics;
- white Gaussian noise;
- sources estimated to contribute to background brain activity.

The reconstructed components are thus a very close approximation of true SSEP signals corrupted only because of possible brain activity that could not be modeled using second order statistics and not common in all the 12 trials. Performance of signal averaging, using the 12 reconstructed components, like the traditional approach can eliminate this

(11) $$\begin{array}{c} X_{r}={\left[\begin{array}{cccc} xr_{1}(t) & xr_{2}(t) & \cdots & xr_{12}(t) \end{array}\right]}^{T}, \end{array}$$

where *xr*_*k*_(*t*) represents the *k*th reconstructed trial with 640 time samples arranged along the row:

(12) $$\begin{array}{c} s\left(t\right)=\frac{1}{12}\overset{12}{\underset{k=1}{\sum }}xr_{k}\left(t\right). \end{array}$$

This signal *s*(*t*) represents the SSEP that remains consistent through the 12 trials used in Section 2.4.2 above. Algorithm steps “Window based preconditioning” and “Eigenspace filtering” are continuously carried out while data acquisition is valid and the surgery is proceeding. Since the algorithm requires only 12 trials to extract an SSEP, it requires only a few seconds as opposed to the conventional signal averaging method. As shown in Figure 3, we notice that this eigenspace filtering is able to (1) remove the spurious frequencies, (2) align the most relevant signal components, and (3) preserve the SSEP morphological characteristics extracted by the Chebyshev window-based preconditioning step.

## 3. Results and Discussion

For IONM using tibial serve SSEP, the peaks that typically occur at 37 ms (positive) and 45 ms (negative) as denoted by P37 and N45, respectively, were monitored. The SSEP obtained just before the surgical procedure is called the baseline signal and was considered as the reference throughout the surgery. It is an established protocol that (1) the time latencies of these peaks should not deviate more than 10% and (2) the peak-to-peak amplitude should not deviate more than 50% from the corresponding baseline values. Any deviation from this protocol is considered a cause for alarm and necessary steps will be undertaken for the safety of the patient.

The algorithm was tested on 12 and the results are presented in Table 1. The algorithm was successful in extracting the SSEP signals throughout all surgeries using only 12 trials that passed the exclusion criterion and checks for the consistencies and any causes for false alarms. The 12 surgical cases did not have any alarms raised during the clinical procedures. The algorithm, however, did raise alarms and these can be termed as “false alarms.” This fact assures us that the algorithm is capable of detecting and raising alarms. As such, the accuracy of the algorithm was defined as

(13) $$\begin{array}{c} \%\text{Accuracy}=\left(1-\frac{\text{no}.\;\;\text{of}\;\;\text{false}\;\;\text{alarms}}{\text{no}.\;\;\text{of}\;\;\text{SSEP}\;\;\text{signals}}\right)\times 100. \end{array}$$

The sensitivity and accuracy of the algorithm can be analyzed based on the number of detections by the algorithm and the actual alarms raised. In the surgeries performed, there were no alarms raised during the surgeries; however, the algorithm did raise alarms that can be understood as false alarms. It was shown in Table 1 that there are, on an average, 1.6 false alarms per hour. If the false alarms are quantized as the percentage of false alarms occurring per subject for every set of 12 trials used to extract the SSEP, we obtain an average of 0.09% of false alarms.

It is very important to note that since the extracted SSEP signals are obtained every twelve trials and hence the short-term SSEP changes, that would have otherwise gone unnoticed by the conventional averaging method easily detected when using the proposed algorithm. Hence, for a true positive, the changes must persist for 12 successive SSEP signals extracted using the algorithm. No such case was observed in the study confirming that no alarms were raised during the procedures.

**Figure 4:**
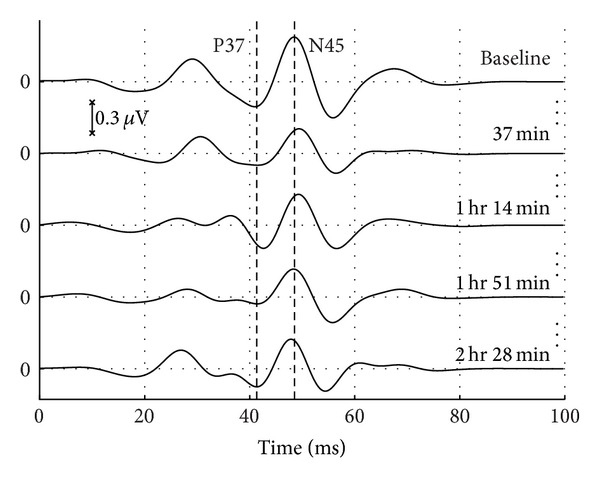
Comparisons of SSEP that were extracted through the surgery with the baseline SSEP at five stages during the surgery of patient number 8.

On an average, the algorithm raised 1.6 false alarms per hour and presented an accuracy of 91.5%. An example of a typical SSEP extracted using the proposed algorithm for a given subject at five different instances of time during the surgery is shown in Figure 4. Note the merits of using Chebyshev as means to preserve the morphology of the SSEP signal.

In our previous publication, it was observed that even though the automation scheme was shown viable, the IIR filtering applied at the very end might not give an SSEP true to its morphological characteristics to be observed by experienced eyes [14]. We observed, in the present study, that such filtering is more beneficial when applied prior to the eigenspace filtering. Conventional systems also adopt a similar approach of filtering using moving average type filters after signal averaging. These are linear phase filters. Hence, we choose Chebyshev time windowing ante eigenspace filtering. This has the merit of limiting power leakage of the frequency components of SSEP to adjacent frequencies. A rectangular window in the frequency domain eliminates undesired frequency components and preserves those frequencies that contribute most to the SSEP signal. It is also effective in removing the 60 Hz noise introduced by the electrical equipment in the vicinity.

It is a long known fact that EEG sources are non-stationary [19]; however, during the 100 ms time windows, as common in IONM, the EEG sources are considered quasi-stationary [20, 21] and hence the AMUSE algorithm was able to identify only the known stationary SSEP component. On the other hand, the use of Chebyshev time window made us certain that any other stationary and quasistationary components are eliminated.

## 4. Conclusions

The current algorithms assume that the SSEP does not change during the time when the 200 or more trials are recorded and rely on the frequency characteristics of the signal rather than the SSEP morphology in time domain. Other proposed approaches also tend to focus on only one aspect of the SSEP characteristics, mainly in the frequency domain. The present approach in retrospect, focuses on preserving the time domain features of SSEP and eliminate the inter-trial variance and extract the SSEP while considering the ongoing brain activity. To ascertain the validity of the algorithm, we present the results of the implementation of the algorithm on 12 surgical procedures that lasted anywhere from 2 to 6 hrs. These surgeries were successful with no resulting neurophysiological disorders. The algorithm also ascertained that the peak latencies and peak-to-peak amplitudes are within the required limits to prove consistency. The algorithm, however, raised 1.6 false alarms per hour. This can be considered as a good sign because this proves that the algorithm is capable of detecting a true alarm, although not experienced in these surgeries. Future study will involve the implementation of this algorithm on patients where a clinical alarm was indeed raised and assess the algorithm's accuracy in detecting the true positives.

## Figures and Tables

**Table 1:** Algorithm implementation and results. Description of the 12 surgical procedures including the procedure they underwent and the time for which the patients were monitored using SSEP and the algorithm implementation results including the accuracy and the number of false alarms detected per hour.

Number	Surgical procedure	Duration (hrs)	Accuracy (%)	False alarms per hour
1	Cerebral aneurysm clipping	2	92.7	4
2	T10-S1 posterior spinal fusion	2	88.1	3
3	Cerebral aneurysm clipping	2	95.9	4
4	T10-S1 posterior spinal fusion	6	94.4	5
5	Anterior and posterior lumbar fusion	4	94.7	4
6	T4-S1 posterior spinal fusion	1.5	91.2	2
7	Posterior spinal fusion for scoliosis	2	89.4	2
8	L5-S1 TLIF	2	96.3	5
9	T10-S1 posterior spinal fusion	2.5	87.2	4
10	T2-T12 post spinal fusion	3.5	95.4	3
11	Carotid endarterectomy	1.4	94.3	3
12	AV fistula	1.2	78.6	2

		Average:	91.5	1.6
